# Selective Manipulation of the Gut Microbiota Improves Immune Status in Vertebrates

**DOI:** 10.3389/fimmu.2015.00512

**Published:** 2015-10-09

**Authors:** Ana Montalban-Arques, Peter De Schryver, Peter Bossier, Gregor Gorkiewicz, Victoriano Mulero, Delbert Monroe Gatlin, Jorge Galindo-Villegas

**Affiliations:** ^1^Institute of Pathology, Medical University of Graz, Graz, Austria; ^2^Laboratory of Aquaculture & Artemia Reference Center, Ghent University, Ghent, Belgium; ^3^Department of Cell Biology and Histology, Faculty of Biology, Instituto Murciano de Investigación Biosanitaria Virgen de la Arrixaca, University of Murcia, Murcia, Spain; ^4^Department of Wildlife and Fisheries Sciences, College of Agriculture and Life Sciences, Texas A&M University, College Station, TX, USA

**Keywords:** fish, host–microbe, humans, immunity, microbiota, prebiotics, probiotics, vertebrates, SCFA

## Abstract

All animals develop in association with complex microbial communities. It is now well established that commensal microbiota is essential for the correct functionality of each organ in the host. Particularly, the commensal gastro-intestinal microbiota (CGIM) is a key factor for development, immunity and nutrient conversion, rendering them bio-available for various uses. Thus, nutritional inputs generate a positive loop in maintaining host health and are essential in shaping the composition of the CGIM communities. Probiotics, which are live exogenous microorganisms, selectively provided to the host, are a promising concept for manipulating the microbiota and thus for increasing the host health status. Nevertheless, most mechanisms induced by probiotics to fortify the immune system are still a matter of debate. Alternatively, prebiotics, which are non-digestible food ingredients, can favor the growth of specific target groups of CGIM. Several metabolites are produced by the CGIM, one of the most important are the short-chain fatty acids (SCFAs), which emerge from the fermentation of complex carbohydrates. SCFAs have been recognized as key players in triggering beneficial effects elicited by simple diffusion and by specific receptors present, thus, far only in epithelial cells of higher vertebrates at different gastro-intestinal locations. However, both strategies have shown to provide resistance against pathogens during periods of high stress. In fish, knowledge about the action of pro- and prebiotics and SCFAs is still limited. Thus, in this review, we briefly summarize the mechanisms described on this topic for higher vertebrates and discuss why many of them may operate in the fish gut representing a model for different mucosal tissues

## Introduction

**Figure 1:**
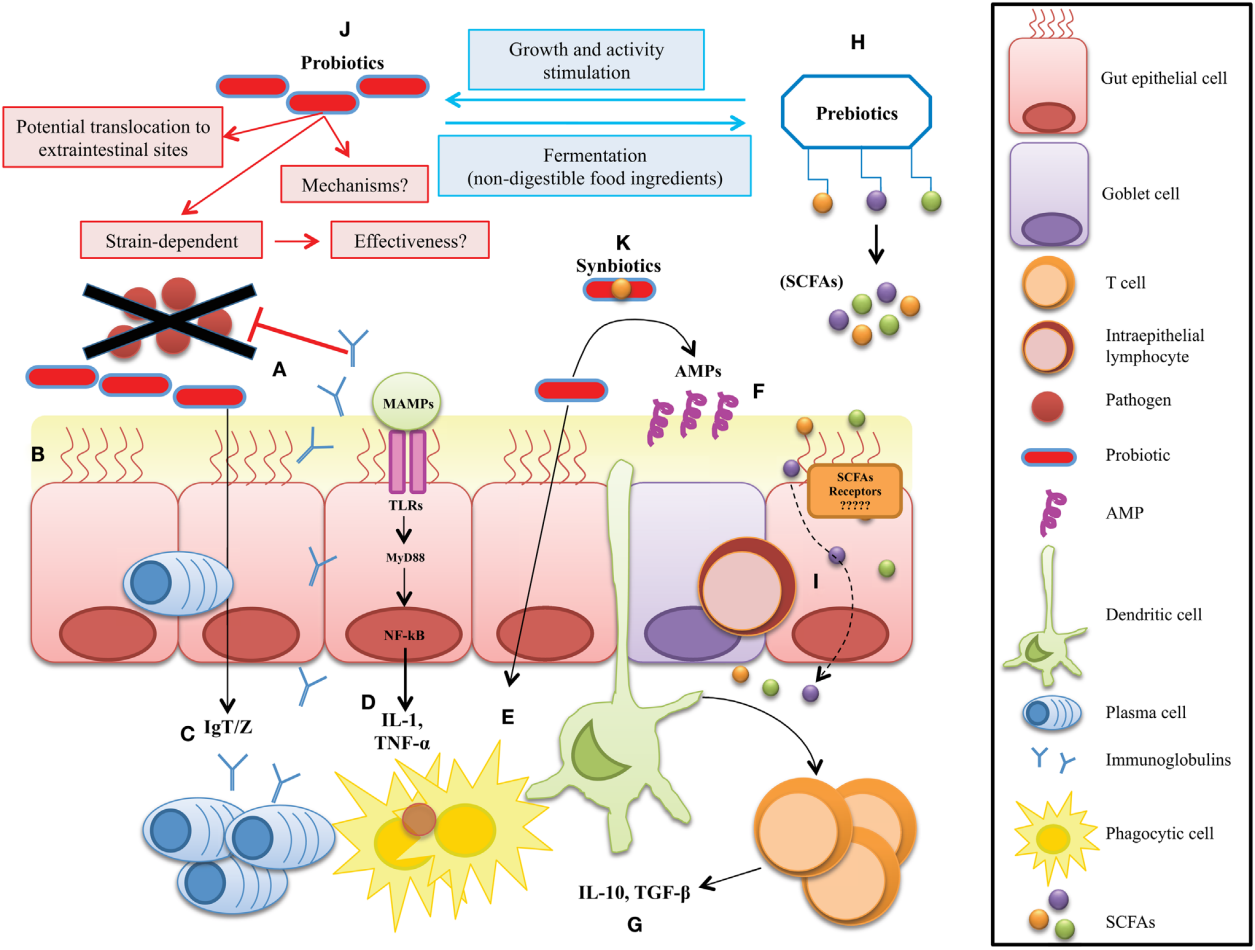
**Potential microbial strategies to improve gut mucosal immunity in fish**. The therapeutic approach mechanisms include: **(A)** competitive exclusion for binding sites and translocation, **(B)** enhanced barrier function by reversing the increased intestinal permeability, **(C)** enhanced mucosal immunoglobulin IgT/Z response to enteral antigens, **(D)** reduction of secretion of inflammatory mediators, **(E)** stimulation of innate immune functions, **(F)** stimulate the release of antimicrobial peptides (AMPs) at the mucosal layer, and **(G)** enhanced availability of anti-inflammatory mediators by regulatory immune cells. **(H)** Production of metabolic health-enhancers like SCFAs by non-digestible prebiotics, **(I)** diffusion of SCFAs through the enterocytes to improve mucosa barrier functions. **(J)** Probiotics have been suggested to confer several health benefits on the host. However, their mechanisms of action are not well understood. **(K)** Synbiotics are a mix of pre- and probiotics, thus their mode of action are much more difficult to define.

**Figure 2:**
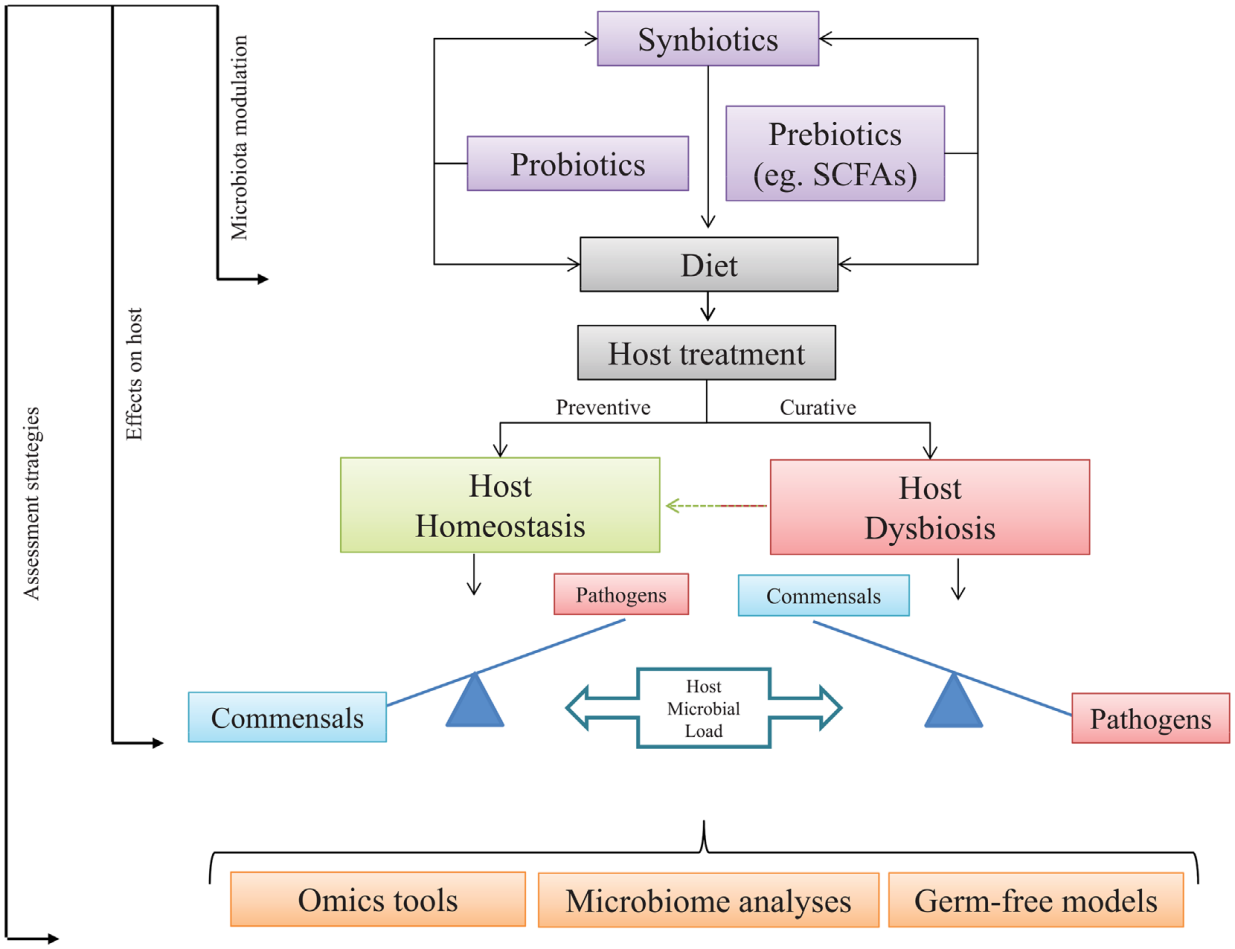
**Host-microbial load under health and dysbiosis**. Addition in diet of exogenous microbial sources may increase fish health through a host–microbe positive loop. Commensal gut microbes might be modulated by dietary administration of target microbes, non-digestible elements, or a mix of both. Expected output should turn in preventive or curative strategies. The use of pro-, pre-, and/or synbiotics is expected to restore the homeostatic stage. Assessment of the selected approach might be quantified, modeled, or dissected using omics tools, germ-free models, and microbiome analyses.

In a broad sense, every single microorganism has been perceived to be a pathogenic threat, which, if uncontrolled, may cause devastating disease in complex live organisms. To understand this view, we only need to look at the latest scientific reports describing the impressive diversity and complexity of the whole microbiome of healthy individuals (1–3). Furthermore, several experimental models ranging from invertebrates to higher mammals, whether conventionally raised (CONR) or germ-free (GF), have provided opportunities to increase our knowledge of the sophisticated nature of the host–microbiome interplay in health and disease, with a level of experimental control hardly achievable in human studies (4, 5). Despite the complexity of studying host–microbe interactions in humans, large scale studies like the Human Microbiome Project or the MetaHit consortium has analyzed the composition of the microbiota of the human body, producing an enormous dataset of bacterial metagenomic and16S rRNA gene information. Therefore, these projects provided also a preliminary understanding of the biology and medical significance of the human microbiota and its collective genes (6). By contrast, in Teleost fish, as the first vertebrate group with full capacities to mount disease resistance strategies through adaptive and innate immune mechanisms, microbiome research lags well behind that for higher taxa. Nevertheless, efforts have been made to reveal the structure of the intestinal microbiome of a few fish species inhabiting a wide range of habitats in recent years (7–10), with the main achievements being attained in the novel host–microbe vertebrate model, the zebrafish (11–13). However, our knowledge of microbiota variations in fish is far from complete, and it is still unclear whether structural microbiome alterations found in diseases, or are just an epiphenomenon. Complex microbial communities colonize virtually every surface that is exposed to the external environment in any organism and include members of the prokaryota, eukaryota, and viruses, which, together provide an enormous enzymatic capacity and play a key role in controlling many aspects of host physiology. However, the core microbiota could be instantly altered, generating dysbiosis. Such dysbiosis represents the state in which the microbiota ecological balance is critically disturbed, triggering perturbations in the meta-community structure, which may damage some of the less represented beneficial species, thereby producing pathological states at any developmental stage of the host (14, 15). For example, antibiotic treatment may lead to diarrhea since pathogen and commensal/mutualist microbes as well are depleted (15, 16). Therefore, in antibiotic-induced dysbiosis, among the several functional aspects affected, the microbiota losses its ability to break down fibers and starches into absorbable short-chain fatty acids (SCFAs), resulting in high levels of undigested carbohydrates, triggering a pathological state in the host (17). Over the past few years, the field of immunology has been revolutionized by our growing understanding of the fundamental role played by the microbiota in the induction, education, and function of the vertebrate immune system, ranging from fish to humans (18, 19). Thus, now it is widely accepted that the microbiome exerts beneficial effects on or within vertebrates to maintain overall health (20). In fish, interrelations between both entities are even more complex since microbes and animals share their outer environment (e.g., water), which is characterized by a high load of organic material, which directly supports microbial growth (21). Therefore, the marked differences between fish and mammals in immune functionality and the intrinsic selective pressures lead to differences in their commensal microbiota. However, it is of interest that the most numerous immune cells in the body of most vertebrates are cells resident at sites highly colonized by commensal bacteria, such as the skin or the gastro-intestinal (GI) tract. This may be a result of the formidable challenge represented by the multiple exposure to microbiota, food-derived antigens, metabolites, and pathogens that require a highly complex network of regulatory pathways in the GI tract (Figure 1), which is only just beginning to be understood (22). Interestingly, among the latest achievements, functional genomics using GF vertebrate models colonized with selected single bacteria or defined consortia show that responses to their compositionally distinct microbial load elicit conserved responses (12). More recently, Seedorf and colleagues (23), using an elegant approach, demonstrate the way in which xenomicrobiota from distant different foreign environments (from soil to human microbes) have the capacity to persist and invade vertebrates with GF guts or presenting established communities, based solely on their capacity to specifically metabolize dietary and host carbohydrates and bile acids. These key experiments clearly demonstrate the importance of the microbiota and the usefulness of GF animals to address a variety of mechanistic questions, suggesting the microbiota-directed therapy as an excellent approach to improve host health. Among the different strategies used to manipulate host–microbe interactions, diet is a major factor that shapes the proportional representation of the microbiota present in the gut and their relative gene content (24). Reciprocally, the configuration of the microbiota influences the nutritional value of food. Therefore, as in mammals, it is tempting to believe in the possibility of achieving a beneficial relationship between microbiota and host health through the manipulation of existing microbial communities by exogenous administration of live microorganisms or non-digestible substrates (probiotics and prebiotics, respectively, or the mix of both called synbiotics) to improve the health status of the host, and to prevent or even cure some preexisting pathologies (Figure 2) (25, 26). In this review, we briefly summarize the above mentioned knowledge obtained in higher vertebrates and propose the administration of SCFAs (27) as an attractive alternative to traditional approaches aimed to improving fish health.

## Host–Microbe Interactions

Multicellular organisms exist as meta-organisms comprised of both the macroscopic host and its mutualistic commensal microbiota. Their coexistence starts at birth, since until that time, all animals are believed to be sterile (28). With an estimated composition of 100 trillion cells, human symbionts outnumber host cells by at least a factor of 10, which express 10-fold more unique genes than their host’s genome (19). Despite the impressive load of microorganisms in the human body, most of commensals are anaerobes belonging only to three phyla: Bacteroidetes, Firmicutes, or Proteobacteria, in order of abundance (29). However, among all the tissues in a vertebrate body, the GI tract is home to a diverse and abundant bacterial community. In humans, the GI community varies, starting at the upper tract with a modest 10^1^ and culminates with an impressive 10^14^ cells/g in the colon represented by ~1000 different species, which have been well characterized (30–32). But, despite this high number of microbes, most symbiotic relationships between hosts and their commensal microbiota, as mentioned above generate several beneficial links (33–36) (Figure 1). For example, vertebrate genomes harbor a very limited repertoire of glycoside hydrolases, and so gut microbes are essential for conferring metabolic traits to extract energy from the fibrous portion of plants with nutritional value (37, 38). In contrast to mammals, it is much more difficult to estimate the total bacterial species present in fish, because factors like habitat, salinity, and trophic level directly shape the microbiota composition (10, 39). Early studies performed in zebrafish demonstrated that, even when reared in different environments, the GI microbiota presents few differences, demonstrating that the co-evolution of fish and their gut microbiota is stronger than the possible influence of the environmental bacteria in shaping these communities (12). Recently, the gut bacterial community censuses in some fish species have been investigated (40–44), and most studies are in agreement that fish GI tract harbor a bacterial load close to 10^8^ bacterial cells g^−1^ represented by ~500 species, consisting mainly of aerobic or facultative anaerobe microorganisms although strict anaerobes are also present (7, 11, 13). At the beginning of the meta-sequencing era, Rawls and colleagues reported for the first time a full list of the main bacterial operational taxonomic units (OTUs) present in the gut of zebrafish (12), and later studies revealed the presence of a core microbiota, dominated by gammaproteobacteria and Fusobacteria (45). Recently, an interesting study of the intestinal microbiome of Asian seabass determined the primary microbial gene catalogcatalogue, investigating the changes in microbial composition of normal and starved fish (46). The authors concluded that Proteobacteria, Firmicutes, and Bacteroidetes are the most abundant intestinal bacterial phyla in Asian sea bass, which, interestingly, are the same phyla as those observed in most fish analyzed to date (39). Therefore, these results reinforce the possibility of microbial manipulation to modify target bacterial groups present in sympatric species. However, host factors like genetic background and gut anatomy may influence the GI bacterial composition (47). For a complete review on the tools used to dissect different fish microbiomes and the species examined so far, we refer to the review of Llewellyn et al., see: Ref. (48). Indeed, the coincidence of most intestinal bacterial communities in fish reveals a conservation status among species that provides fish with the same capacities to survive in the aquatic environment. Surprisingly, six of the eleven bacterial divisions found in adult zebrafish are also found in mice, and five are also shared by the adult human microbiota (12). These diverse microbial conformations among species, as analyzed by functional genomics, point to host responses shared with their compositionally distinct microbial communities that elicit conserved responses. New omics technology will permit the composition of specific microbial communities in any species to be evaluated and establish co-evolutive links among host and microbes, opening up the possibility to determine the balanced and reciprocal outputs produced among them under diverse scenarios (e.g., after microbial manipulation). Thus, we anticipate that fish microbiome research will make significant progress in the mid-term. To date, when GI microbes have been compared between mammals and herbivorous fish by UniFrac, they have been seen to cluster relatively close, perhaps due to a common gut fermentation process that leads to the production of SCFAs (39). This would suggest that although taxonomic composition at a particular body site can differ dramatically from one individual to another, functional composition measured from bacterial RNA data is well conserved. However, when focusing on RNA transcripts, it has been observed that variation depends on a subject-specific environment, which can be influenced by both long- and short-term regulatory changes in the gut (49). These results emphasize that fish commensal microbe composition is not a simple reflection of the microorganisms in their local habitat but may result from host-specific selective pressures within specific tissues, thereafter highly colonized by microbes, like the gut or skin in their different developmental stages (22). The impressive plasticity of the fish GI microbiota suggests that selective manipulation of microbes or their sub-products may produce health benefits without altering intestinal morphology or physiology.

## Germ-Free Animal Models

To analyze gene expression (metagenomics), protein products (metaproteomics) and metabolic profiles (metabolomics), GF animals emerge as an indispensable tool, which may help to decipher the microbiome composition, metabolic activity, and mechanisms used by microbiota in health and disease (50). This tool has already greatly contributed to understand the development of the vertebrate immune system, physiological processes, and the mechanisms responsible for inflammatory and neoplastic diseases (18, 51–54). Gnotobiotic models, a variant of GF models, are organisms with a defined microbiota, which can range from a single bacterial species to a known consortium, introduced alone, together, or sequentially in the host. Most GF experiments are performed with mice or rats, but rabbits, pigs, and fish among other species have been used as well. Therefore, there is much evidence on the crucial role of the microbiota in several physiological functions of vertebrates, among them: the priming of neutrophils and increased disease resistance against viral infection (18), the initiation and progression of inflammation-associated colorectal neoplasia (50), regulation of the intestine antimicrobial RegIII by the probiotic species *Bifidobacterium breve* NCC2950, but not by the commensal *Escherichia coli* JM83 (55), increased susceptibility to arthritis due to deficiencies in reactive oxygen production (56), modifications in the properties of the gut mucus barrier through the lack of sufficient Protobacteria after transfer of cecal microbiota to GF recipients (57), or central changes in brain expression after feeding peptides (58). Consequently, comparative studies using GF models have demonstrated themselves to be fundamental for characterizing the profound interactions between the microbiota and the host. Interestingly, GF animals have shown defects in both the development of the immune system and in immune responses due a reduced intestinal vasculature, undeveloped gut-associated lymphoid tissue (GALT), altered energy harvest (fecal caloric content), storage (weight and body fat), and expenditure compared with animals housed under specific pathogen-free (SPF) conditions (59). Therefore, it is not surprising that in the last decade, some interesting reports on the generation or usage of different GF fish models have been reported (9, 12, 18, 60–63). Interestingly, in most of these reports, GF organisms trend to be smaller than age-matched SPF organisms, and demonstrate reduced anxiety-like behavior, which may be linked to metabolic changes due to the absence of microbiota. However, all along the vertebrate lineage, it has been reported that most features, including the immune status, are largely restored once the microbiota are reestablished (11, 13, 18, 64). Therefore, despite the many difficulties still to be solved in the maintenance of GF fish, this biotechnology can be regarded as a powerful tool to address a multitude of questions about the coexistence of vertebrates with microorganisms and point to the importance of microbiota in the establishment of functional immunocompetence.

## Basic Features of the Immune System

The innate immune system provides a first line of defense against microbes through primary responses, which includes phagocytosis and the induction of inflammation. Immune-competent cells play a primary role in recognizing microbial-associated molecular patterns (MAMPs), which are diverse molecules not present in any of their structures, and include various molecules ranging from lipoproteins, lipopolysaccharide (LPS), flagellin, and peptidoglycan to unique bacterial nucleic acid structures, such as cyclic dinucleotides (CDNs) in several microbial types (65). These MAMPs are mainly recognized via pattern-recognition receptors (PRRs) that are expressed in all cells of a given type and grouped into two well-studied families with wide recognition capacities: the transmembrane Toll-like receptors (TLRs) and the intracytoplasmic Nod-like receptors (18). A wide set of TLRs have been reported in mammals (66), while in fish, a complete set of TLRs seems to be functional orthologs of their mammalian counterparts (67) (Figure 1). Surprisingly, recognition capacities, not simply as the commensal microbial partners, are known to have the same conformational, molecular, or locomotive structures as the pathogens. Therefore, huge efforts should be entitled by immune cells to initiate antimicrobial defense mechanisms mediated by several conserved signaling pathways among vertebrates, as illustrated in several recent reviews (68–71). The activation by PRRs of key master regulators, such as the nuclear factor-κB (NF-κB) and interferon-regulatory factors, promotes the production of powerful antimicrobial molecules, such as inflammatory cytokines (72) or interferons (73), respectively (Figure 1). However, if the threat remains, cytokines and interferon will instruct the immune response to switch toward a specific mode of action mediated through adaptive immune recognition. This specific response is mediated by two types of antigen receptor: T- and B-cell receptors. The genes encoding antigen receptors are assembled from variable and constant fragments through recombination-activating gene (RAG) protein-mediated somatic recombination, a process that yields a diverse repertoire of recognizing proteins (74). This diversity is further increased by additional mechanisms, such as non-templated nucleotide addition, gene conversion, and (in the case of B cells) somatic hypermutation, generating a high diversity of receptors with the potential to recognize almost any antigenic determinant in a specific manner (75). Of particular importance in fish, as in mammals, is T-cell activation and induced proliferation, which leads to a T-cell-mediated response via cytotoxic lymphocytes located at mucosal surfaces, such as intraepithelial lymphocytes (IELs) (76, 77). The development of memory allows the immune system to maintain a B-cell profile corresponding to a specific pathogen, which in the face of a second infection will proliferate quickly to fight it (77, 78). Although these adaptive mechanisms are present in fish, memory achievements are much less developed than in mammals. However, paradoxically, apart from the recognition mechanisms, vertebrates have developed the means to tolerate large populations of microbial partners to preserve homeostasis during their life cycles. This fine borderline separating microbiota, health, and disease has led to an increased interest in the study of host–microbe relations. Interestingly, in mammals, it has been reported that, in addition to classic innate immune receptors, specific receptors with capacities to interact with microbial metabolites are present on immune surveillance cells (see section SCFAs as Key Immune Modulators). In fish, the presence of such receptors which recognize specific microbial metabolites deserve further experimentation.

## Mucosal Immunity

In any host, immunity acts at different levels. Among them, all metazoans have a mucosal epithelium, which is one of the oldest and most universal modules of innate immunity. Together with the skin, the mucosal epithelium is the main interface between the host and the microbial world (including both pathogenic and symbiotic microorganisms). Therefore, it has important functions in protecting the host from pathogen invasion and in establishing symbiotic relationships with the host microbiota. Accordingly, as presented in Figure 1, mucosal epithelial cells and skin keratinocytes have specialized antimicrobial functions: for example, the production of antimicrobial peptides (AMPs), which limit the viability and multiplication of pathogens and symbiotic microorganisms that colonize these sites. The key players at the mucosal surface are the mucin-producer cells, which keep microbes at bay and influence microbiota distribution and content (75). In mammals, two secretory intestinal epithelial cells (IECs) lineages, namely goblet and Paneth cells, are critical for this function. Goblet cells form a physical and chemical defense barrier by producing transmembrane mucin glycoproteins and by secreting mucins – notably Muc2 – that cover the intestinal epithelium and form a two-tier inner and outer layer to prevent bacterial adhesion to the epithelium (79). Additionally, goblet cells also express AMPs sequestered in the mucinous gel (80). While the inner dense mucous layer restricts bacterial penetration and growth, the extended outer layer forms a well-suited environment for resident bacteria (81). More particularly, the importance of mucous protection in gut homeostasis is demonstrated by the development of spontaneous colitis in Muc2-deficient mice (82) and by the reduction of goblet cell numbers and depleted mucous secretion in inflammatory bowel disease patients. Glycosylated mucin proteins are metabolized by specialized mucous-degrading enzyme-producing bacteria. Therefore, released oligosaccharides are used as a food source for the growth of specific bacterial subsets – notably *Bacteroides fragilis* and *Akkermansia muciniphila*, among others. Thus, mucin levels in host are not only bacterial containers, but could affect the abundance and distribution of defined intestinal bacterial subsets (83). In contrast to higher vertebrates, mucosal tissue dendritic and M cells in fish are not well established (84), and Peyer’s patches, mesenteric lymphoid nodes, IgA and J-chain have not been reported [for complete information on fish mucosal immunology, see Ref. (68, 70, 85, 86)]. However, the two specialized mucosal immune molecules which have been unequivocally recognized in fish are worth to be emphasized here due to their high homology with their mammalian counterparts. The gut-specific immunoglobulin IgT/Z (77, 87–92) and the molecular identities of skin and gut mucins, like Muc2 (93–95). Thus, if at least IgT and Muc2-dependent mucus production are critical for effective management of both pathogenic and non-pathogenic bacteria, the induction at the GI tract of these key molecules through exogenous sources in the mucosal tissue (96, 97) is a promising therapeutic alternative in most vertebrates without excluding fish.

## Diet as a Tool to Modulate the Intestinal Microbiota

Powered by novel technologies and major international initiatives, host–microbe research activity has transformed our understanding of the gut microbiota, including its interactions with diet and health. Multiple studies have shown that by virtue of their catalytic activity, the microorganisms in any vertebrate play a critical role in shaping the gut microbiota, GI function, immune regulation, and host health (98, 99). Reciprocally, our increasing knowledge of the characteristics, ecology, and composition of the gut microbiota has intensified interest in modulating the gut ecosystem. Several studies using diverse vertebrate models like chickens (100), swine (101), mice (102), humans (103, 104), and fish (105–109) have shown the possibility of applying dietary strategies to modulate the commensal gastro-intestinal microbiota (CGIM). Most studies suggest that the conversion of dietary components by intestinal bacteria leads to the formation of a large variety of metabolites, which may have beneficial or if uncontrolled adverse effects on vertebrate health (Figure 1). This observation is clearly illustrated in humans by a comparison of the incidence of inflammatory and metabolic diseases, such as type II diabetes, colon cancer, and asthma associated with “Western” diets and the low prevalence of the same associated with diets rich in dietary fiber, as found in rural areas (110, 111). Among the most recurrent GI disorders in the industrialized world is irritable bowel syndrome (IBS), which is present in about 10–15% of the population and despite it being a multifactorial pathology, most patients report their symptoms to be triggered by meals or specific foods. This, together with a known dysbiosis linked to IBS, provides evidence regarding the impact of diet, the intestinal microbiota, and their inter-relation in the development of the disease (112). Despite representing almost half of the total number of vertebrates, less attention on this type of host–microbe studies has been done in fish (39), as described at the previous sections. However, due to fish appearing much earlier in evolution, their study could help us understand much better the host–microbe co-evolution and therefore their functional importance on diet processing in higher vertebrates. In this regard, impressive advances have been observed in the development of functional feeds/diets. The term “functional feeds” is used to describe a particular type of food/feed that has added benefits that will improve both health status and growth promoting performance of the animals which ingest them, mainly by supplying additional compounds above and beyond the basic nutritional requirements for animal growth alone (113). Thus, this approach enables a significant shift away from chemotherapeutic and antibiotic treatments. In addition, the development of new feeds or diet strategies to ensure that both fish and the final product are of the highest quality (114) and replacement of fishmeal and fish oils with vegetable products is now being partially achieved (115). Unfortunately, there has been limited study of the health aspects of the newly designed feeds, an area that will have much more attention in the future (116–118). Therefore, we could say now that responses to these new diets are still far from elucidated and only some altered genes and proteins in different tissues of the fish have been reported (119–121). Nonetheless, a selection of several additives is available for inclusion in functional feeds. Among them are the immunostimulants, which will not be discussed in this review since they directly impact immune receptors rather than the host–microbe axis, as opposed to probiotics, prebiotics, synbiotics, and ultimately the CGIM metabolites.

## Probiotics

For many years, several bacteria and yeasts have been regarded for their health-inducing properties. The term probiotic was initially defined as live microorganisms which when administered in the diet in adequate amounts confer a health benefit on the host. The definition differentiates live microbes used as processing aids or sources of useful compounds from those that are administered primarily for their health benefits. A distinction between commensal microorganisms and probiotics is also inferred from this definition. Although commensals in the gut are often the source of probiotic strains, until these strains are isolated and characterized and a credible case is presented for their health effects, they cannot be called “probiotics” (122). But, recent findings have thrown light on previously unknown aspects. Among them, several concerns regarding efficacy, like the absence of substantiated health claims, the use of fecal microbiota transplants outside of the probiotic framework, or the inclusion of native colonizing microbes of the host with adequate safety and efficacy, have provided recent updates to the probiotic concept (122). So far, the most widely investigated microorganisms that display adequate probiotic characteristics are those from the genera *Lactobacillus* and *Bifidobacterium* (123). However, several other commensals, such as *Propionibacterium*, *Streptococcus*, *Bacillus*, *Enterococcus*, *E. coli*, and yeasts also have been investigated in this respect (124). However, no matter how effective they are, one concern associated with all the microorganisms proposed as probiotics to date is the large number of active proteins intimately associated with them, which make it difficult to define their mechanisms of action. Amid such considerations is the idea that if mechanisms are not always well understood, their effectiveness may be in doubt, which is a solid reason for suggesting that using probiotics in humans or animals should be carefully considered. Nevertheless, research has encouraged speculation on how they work. For example, as proposed in Figure 1, they may revert dysbiosis and normalize the host microbiota, inhibit adhesion of pathogens to the epithelium, increase production of mucin and AMPs, strengthen the mucosal barrier, or modulate the immune system through enhanced cell-mediated responses [for a complete review on proposed modes of action in mammals and fish, see Ref. (125, 126), respectively]. In fish, the use of probiotics has also been found to stimulate immunity (127), although the exact mode of action, that is, at the basis of these observations remains unknown. To define mechanisms in fish, it is much more complicated due to their extensive taxonomical and ecological variations, which depend on the fish species and bacterial strains used. Hence, not all strains are beneficial for all disorders or for all species, and some may be detrimental to some hosts, or even worse, they may aggravate health problems if these already exist (128). Thus, a probiotic must at least have the capacity to survive in the GI tract, display high resistance to gastric acids, lack any transferable antibiotic resistance gene, and have the capacity to exert clear benefits in the host through the modulation of the resident CGIM. Moreover, they should be non-pathogenic, non-toxic, and provide protection against disease-causing microorganisms by means of multiple conserved mechanisms (45). These properties have been clearly dissected in many excellent reviews on probiotics. However, we will simply mention a recent finding, which may give light to specific mechanisms by describing how only a small fraction of the intestinal microbiota (as scarce as a single bacterial species) may confer infection resistance. Buffie et al. (129) described the synthesis of *Clostridium difficile*-inhibiting metabolites from host-derived bile salts by CGIM members. Therefore, the use of a human-derived *Clostridium scindens* isolate to augment murine *C. difficile* inhibition emphasizes the conservation of this finding across species and suggests therapeutic and diagnostic applications for a wide range of vertebrate species. Unfortunately, contrary to the stable and beneficial effects observed in higher vertebrates, the output of probiotics as modifiers of the CGIM in fish until now is quite variable (130). Therefore, different approaches with more refined compounds should be explored in fish.

## Prebiotics

A prebiotic has been defined as an ingredient selectively fermented by specific health-promoting bacteria that allows specific changes both in the composition and the activity of the GI microbiota by increasing the release of bacterial metabolites that confer benefits upon host well-being and health, while inhibiting the growth of pathogenic bacteria (126, 131, 132). Currently used prebiotics are mainly poorly digestible carbohydrates with a relatively short-chain length classified on the basis of their molecular weight (126, 133). According to their degree of polymerization, prebiotics are classified into mono-, oligo-, or polysaccharides (134). But, based on their physiological and biochemical properties, the carbohydrates can be classified as digestible or non-digestible (135). Modulation of the gut microbiota by means of non-digestible carbohydrates is more common than using the digestible fraction, since they may exert several effects mediated through different metabolic pathways, including glucose and lipid metabolism, inflammatory reactions, and even changes in appetite regulation (136). In mammals, the administration of prebiotics, which include inulin-type fructans, fructo-oligosaccharides (FOS), galacto-oligosaccharides (GOS), xylo-oligosaccharides (XOS), lactulose, and human milk oligosaccharides have received attention and several beneficial effects have been reported (137–139). Such benefits include reduced intestinal low-grade inflammation, improved gut barrier integrity, the production of anti-inflammatory mediators, reduced blood cholesterol, and improved food assimilation or immune cellular components and mucosal barrier fortification (140–142). Therefore, following the successful trend observed in endothermic animals, several studies have proposed that feeding prebiotics, purified or mixed with different compounds, might be beneficial to both freshwater and marine cultured fish (106) [for full reviews, see Ref. (126, 128)]. However, investigations in some fish species have revealed that such an assertion is only partially true and caution must be taken when administering functional foods. In support of the previous statement, experimental dietary administration of inulin in rats, chickens, and humans has proven effective in reducing the adverse impacts of intestinal dysbiosis, such as oxidative dysfunctions and neurotoxicity (143) or the atrophy of lymphoid organs (144). Additionally, it has been suggested that inulin improves CGIM (145), produces several positive physiological effects (146), acts as an anti-obesogenic (136), prevents cardiovascular diseases (147), or even displays anti-cancer properties (143, 144, 148). Therefore, it has been proposed as a promising immune enhancer in fish, but to date, the reported effects have been variable and definitive conclusions are hard to reach (149, 150). Thus, as with inulin, after extensively analyzing the use and effect of several prebiotics in aquaculture fish, it is possible to establish that prebiotics can activate the innate immune system directly or by association with “good” microbes, but caution is needed as the contribution of the molecular mechanisms output will depend, for example, on the compound used, the species, season, and dose because CGIM may vary in different cycles. Therefore, we suggest the use in combination pro/prebiotics or alone of more refined compounds resulting from the fermentation of prebiotics by the CGIM, such as SCFAs, which have been linked with numerous health benefits *in vivo* (151), and may produce a strong and reliable health increase in the host.

## Microbial Metabolites

Ursell and colleagues (152) recently stated that most human gut microbial communities metabolize dietary ingredients in different ways and produce different metabolites that, in turn, can affect the host in several manners. However, the understanding of the molecular mechanisms behind their effects remains low (153). The use of high-throughput metabolomics has potential in this respect. Metabolic analysis of the gut environment comprises the metabolic profiling (identification and quantification) of the repertoire of thousands of small and large molecule metabolites present in the GI tract using high-throughput analytical methods. Popular approaches are the use of methods based on mass spectrometry (154) and nuclear magnetic resonance spectroscopy (155, 156). However, the complete set of metabolites has a highly variable chemical structure and properties and results depend on the host’s physiological state (157). Microbes provide the host with a rich range of metabolic capabilities. The availability of spectral networking platforms combined with open-source metabolome databases, such as HMDB, METLIN, LIPIDS MAPS, MassBank, and NIST, allows the identification and annotation of known and unknown metabolites from the metabolome spectral profiles (152). Most metabolomic studies, however, have focused on relating gut microbiota functionality with metabolic outcomes of the host by characterizing molecule metabolites in different host tissues like feces, plasma, urine, or other tissues (158). As such, Wikoff and colleagues found that many plasma metabolites consisting of hydrophilic carbohydrates, volatile alcohols, ketones, amino and non-amino organic acids, hydrophobic lipids, and complex natural compounds (157), were unique to CONR mice as compared with GF mice and that the relative signals of more than 10% of the common metabolites differed significantly between the two. In other studies on humanized mice, metabolomics have been applied to show that probiotic, prebiotic, and symbiotic induced changes of the gut microbiota modulate the host lipid, carbohydrate, and amino acid metabolism at a panorganismal scale (159). The change in the systemic metabolite profile resulting from gut microbiome modulation is, thus, not limited to the gut but also includes other tissues. This illustrates that major metabolic processes are under symbiotic homeostatic control. Fewer studies have focused on the effect that gut microbiota manipulation has on the gut or fecal metabolome. One example is the study of Respondek and colleagues (160) in which the effect of short-chain FOS on the fecal metabolome was evaluated, showing an increased presence of conjugated fatty acids and a decreased presence of bile acid derivatives. Metabolomic studies applied to fish are few, and almost all focus on the influence that dietary treatments, toxicologically active compounds, or culture conditions have on the metabolite composition of different fish tissues (161–167). As far as known, only one study has assessed the gut metabolite profile in fish. The aim of Asakura and colleagues (168) was to investigate gut metabolic variations associated with fluctuations in microbial composition and structure in diverse fish species and to evaluate the effect of changing feed type on the co-metabolic modulations in a fish microbial symbiotic ecosystem. Fecal samples were used for this purpose. Using principal component analysis (PCA) – thus focusing on the overall metabolite profile rather than specific metabolites – they found that metabolite profiles of fish of the same species clustered together under natural conditions, but clustered according to feed type across species when cultured under controlled conditions. It was also observed that the metabolite profile differed significantly between gut content and fecal samples, suggesting that gut content samples provided information on the actual metabolite profile in the gut environment, whereas fecal samples – in general lower in metabolites – were indicative of absorption of the gut metabolites by the fish. As a consequence, the latter sample type does not seem suitable for providing information on the metabolites produced by the gut microbiota. Overall, it can be concluded that the effect of probiotics, prebiotics, or synbiotics on the metabolite profile in a fish gut, or even other tissues, has not yet been investigated in depth. Therefore, much research is essential to substantiate knowledge on how gut microbiota manipulation may be applied to promote immunity and fish health.

## SCFAs as Key Immune Modulators

The gut microbiome can be regarded as a metabolically active organ and modulation thereof by probiotics or prebiotics is becoming increasingly recognized as an important therapeutic option (107, 169). As mentioned above, one of the primaries aims of probiotic, prebiotic, or synbiotic treatments in mammals and fish is the altered production of microbial metabolites. More specifically, the fermentation of prebiotic carbohydrates that escape digestion in the upper GI tract, leading to the production of SCFAs – 1–6 carbons in length, mainly acetate, propionate, and butyrate – by intestinal microbes is targeted to stimulate the health of mammals (159) and also fish (107, 170). SCFAs are the main energy sources of gut cells and as such play a central role in the physiology and metabolism of the gut (171). Gut cell proliferation, cell differentiation, apoptosis, mucin production, lipid metabolism, etc. all seem to be largely mediated by SCFAs. In mammals, however, it is being realized that, besides their function as energy sources for epithelial cells (172), SCFAs are also potential immunostimulatory molecules (98, 173–176), improve lymphocyte function (19, 177), and have immune-related effects resulting from their binding to the G-coupled protein receptors GPR41, GPR43, and GPR109A (178). GPR43 recognizes acetate, propionate, and butyrate and is highly expressed in neutrophils, macrophages, and monocytes; whereas, GPR41 expression is low or undetectable in the same cells (98). Immune-related effects of SCFA recognition include the modulation of anti-inflammatory responses, intracellular cyclic adenosine monophosphate (cAMP) levels, calcium levels, and ERK1/2 activation. For example, Maslowski et al. (179) found that GPR43 stimulation by SCFAs was necessary for the normal resolution of inflammatory responses in mice. Other immune regulatory activities of SCFAs include the inhibition of histone deacetylases (180, 181), regulation of autophagy (182), regulation of T cell differentiation (174), and stimulation of heat shock protein production (183). Although the full spectrum of molecular mechanisms by which SCFA regulate the development and functioning of immune cells remains far from known, it is clear that SCFAs play a central role in mammal immunity. Evidently, the same possibility holds true for fish. In fish, most PRRs homologous of higher vertebrates are present, and the beneficial effects described so far for SCFAs as the end products of microbial fermentation (184) are comparable to that observed in higher vertebrates, although, as far as we know, the immunomodulating potential of SCFAs for fish has not been considered. Specific receptors for SCFAs in fish cells, for example, have not yet been described in the literature. Using the HomoloGene tool of the National Center for Biotechnology Information (NCBI) (185), gene orthologs of GPR41 and GPR43 in mammals can, however, be found in the genome of zebrafish. These include the free fatty acid 3 (GPR41)-like gene LOC100333904 (55.1% gene similarity with FFAR3 from *Homo sapiens*), and the free fatty acid 2 (GPR43)-like gene LOC100004095 (56.5% gene similarity with FFAR2 from *H. sapiens*). As such, it is likely that SCFA can stimulate immunity in fish in similar ways as they do in mammals. For example, the prebiotic bacterial storage compound poly-(-hydroxybutyrate (PHB) (186) has been shown to provide protection against pathogenic infections for a large variety of aquaculture animals including the protection of Nile tilapia against *Edwardsiella ictaluri* gly09 infection. It was suggested by Suguna et al. (187) that this compound acts as an immunostimulator in tilapia although the mechanism is not known. Based on the review of Dedkova and Blatter (188), which describes (-hyroxybutyrate – the monomer and GI degradation product of PHB – as a ligand for GPR41 and GPR109 in mammals, it can be hypothesized that PHB acts in an immunostimulatory way in fish through its SCFAs. We suggest that the *in vivo* identification of SCFAs receptors in fish cells should be a primary target of future research to elucidate the immunomodulating effect of probiotics and prebiotics in fish.

## Concluding Remarks

From the information presented in this review, it is quite clear that, starting from birth or hatching, the presence of microorganisms within any vertebrate, from fish to humans, plays a significant role in the development of immunity and further capacities on disease resistance and health status along life. Among host commensals, the CGIM is of particular importance because any dysbiotic event at this important niche may have profound physiological and metabolic consequences at local and systemic levels (189). Therefore, any alteration on the host–microbe load may lead to impaired responses at different levels, mostly at the immune component that ultimately would lead to disease. Thus, the modulation of the microbial communities in the intestine through dietary administration of probiotics, prebiotics, or synbiotics emerges as a potential strategy to improve microbial metabolite production, the immune signaling pathways, and the host defense mechanisms against pathogens. Consequently, it is of vital importance to deepen or knowledge in the microbial profile present in specific species in particular conditions. This could be achieved by using powerful assessment strategies, like state-of-the-art omics tools, in either microbes or hosts, microbiome analyses which may lead to unequivocally identify the composition of the inhabitants of the GI tract and test hypothesis through the use of GF models. We suggest that following the proposed steps presented in Figure 2, in particular fish species may lead to improvements in host well-being and health, such as those already observed in higher vertebrates.

## Conflict of Interest Statement

The authors declare that the research was conducted in the absence of any commercial or financial relationships that could be regarded as a potential conflict of interest.
